# Removal of Spontaneously Fractured Leads with Their Proximal Ends in the Heart and Vasculature—Description of Different Approaches and Tools

**DOI:** 10.3390/jcm14010282

**Published:** 2025-01-06

**Authors:** Andrzej Kutarski, Wojciech Jacheć, Radosław Pietura, Marek Czajkowski, Paweł Stefańczyk, Jarosław Kosior, Sebastian Sawonik, Dorota Nowosielecka

**Affiliations:** 1 Department of Cardiology, University Hospital of Lublin, 20-059 Lublin, Poland; a_kutarski@yahoo.com (A.K.); s.sawonik@wp.pl (S.S.); 2 2nd Department of Cardiology, Faculty of Medical Sciences in Zabrze, Medical University of Silesia, 40-055 Katowice, Poland; wjachec@interia.pl; 3 Department of Radiography, Medical University of Lublin, 20-059 Lublin, Poland; radoslawpietura@gmail.com; 4 Department of Cardiac Surgery, Medical University of Lublin, 20-059 Lublin, Poland; mczajkowski@interia.pl; 5 Department of Cardiology, The Pope John Paul II Province Hospital of Zamość, 22-400 Zamosc, Poland; paolost@interia.pl; 6 Department of Cardiology, Masovian Specialistic Hospital of Radom, 26-617 Radom, Poland; jaroslaw.kosior@icloud.com; 7 Department of Cardiac Surgery, The Pope John Paul II Province Hospital of Zamość, 22-400 Zamosc, Poland; 8 Institute of Humanities and Medicine, Academy of Zamość, 22-400 Zamosc, Poland

**Keywords:** transvenous lead extraction, fractured lead removal, femoral approach, methods of fractured lead removal, extraction of migrated leads

## Abstract

**Background:** Removal of spontaneously fractured leads with their proximal ends migrated into the vascular space has not been analysed in detail thus far. The study aimed to compare the effectiveness of different approaches and auxiliary tools for removing fractured leads with migrated proximal ends. **Methods:** Retrospective analysis of 72 cases from a database containing 3847 TLEs (transvenous lead extraction). **Results:** Most of the leads were passive, especially unipolar. Procedure complexity in such cases was high but with satisfying effectiveness (procedural success rate 93.06%) and independent of the position of the proximal end. The rate of major complications was 2.78%, which may be attributed to long implant duration (152.2 months). Extraction of such leads did not influence long-term survival. The femoral approach was most often used (62.50%). In 79.16% of leads, mechanical dissection was required. In 66.7%, proximal ends were strongly attached to the wall, and a loop had to be applied. In 15.28% of procedures, the lead was wrapped around a pig-tail catheter (“spaghetti twisting technique”). **Conclusions:** (1) Spontaneous lead fracture with the proximal ends migrated into the vascular space is a rare finding (1.87% of the TLE). (2) Removal of such leads requires the use of different approaches as well as dedicated and non-dedicated tools. (3) Despite a high level of procedure complexity, its effectiveness is high, with an acceptable rate of major complications.

## 1. Introduction

Incorrect lead implantation (too parasternal puncture with subsequent crush syndrome), improper fixation of the cut abandoned leads, and lead fractures due to too tight ligature cause the proximal lead ends to migrate into the vasculature and move further [1,2,3,4,5,6,7,8,9,10,11,12,13,14,15,16,17,18]. Leads with migrated proximal ends (LMPE) to the subclavian or anonymous vein [1,7,8,10,11,12,13,14,15,16,17] or superior vena cava [1,10,11,12,13,14,15,16,17] promote the formation of loops that may pass through the tricuspid valve to the right ventricle and cause tricuspid valve dysfunction [1] and ventricular arrhythmias [8]. Free LMPEs, without encapsulating scar tissue, may float into the pulmonary artery [1,2,5,7] and cause pulmonary embolism [1,2]. Due to the significant risk of these secondary consequences, LMPEs are class 1 or 2b indications for extraction according to the guidelines of the Heart Rhythm Society (HRS) [17,18]. Migrant proximal lead ends have been described in several case reports [3,4,5,6,7,8,9,10,11], and several publications [1,2], as well as lead management guidelines [17,18]. To date, no one has described the techniques and comprehensively analysed their effectiveness in LMPE removal.

It should be noted that conservative treatment options were described, consisting of not undertaking any intervention and recommending further observation of such a patient [3,4,5].

Having a large database of extraction procedures (3847), we set out to compare the different ways fractured leads without accessible proximal ends had been removed and to examine the outcomes of patients undergoing the extraction procedure, taking into account the anatomical conditions.

### Goal of the Study

Our study aimed to compare the effectiveness of different approaches and auxiliary tools for removing fractured leads with LMPE. We decided to describe in more detail the techniques for removing such leads using different approaches and dedicated tools, taking into account the position of the proximal end of the lead and the degree of its immobilization in encapsulating scar tissue.

## 2. Methods

### 2.1. Study Population

A database of 3847 transvenous lead extraction (TLE) procedures performed between March 2006 and March 2023 at three high-volume centres was reviewed to identify patients eligible for the study. Patient clinical characteristics, CIED (cardiac implantable electronic device) characteristics and history of pacing, targeted lead characteristics, extraction complexity, efficacy, and outcomes were retrospectively analysed using our computerized database. For the purpose of this study, 72 patients having LMPEs in the vascular space, which were unavailable in the generator pocket and at the site of lead entry, were selected from the entire database for further analysis. We focused only on this subpopulation.

### 2.2. Definitions

Indications for lead extraction, procedure effectiveness, and complications were defined according to the lead management recommendations (2009 and 2017 HRS consensus) [17,18]. The effectiveness of the extraction procedure was expressed as the rate of procedural and clinical success [17,18]. Major complications during TLE were defined as life-threatening complications that caused significant or permanent disability or death or required surgical intervention (emergency cardiac surgery to prevent procedure-related death or permanent disability) [17,18]. The complexity of the procedure was expressed as total lead extraction time and average single lead extraction time, as well as the use of second-line and advanced tools [19,20]. The third complexity marker was The Complex Indicator of the Difficulty of the TLE, which includes time for extraction of all leads >20 min (2 points), the average duration of single lead extraction >12 min (2 points), use of metal sheaths or Evolution/TightRail, alternative approach, or the need to use lasso catheters or basket catheters (one point each). The sum of the points was the value of CID-TLE [19].

Unexpected difficulties during the extraction procedure (so-called “technical problems”) were defined as unforeseen situations that increased the complexity of the procedure but were not complications. They included blockage at the site of lead entry/subclavian region preventing advancement of a polypropylene catheter into the subclavian vein, Byrd dilator collapse/fracture, lead-on-lead adhesion, use of alternative approach, loss of fractured lead fragment (when the main part of the lead was dissected and removed, but a free mobile piece of the lead remained in the vascular space, usually floating into the pulmonary circulation) and dislodgement of functional leads [20].

Assessment of procedure complexity was based on the MB score (Mazzone–Bontempi score)—showing the need to use advanced tools to achieve TLE success (0–5 points) [21], LED index—referring to lead extraction difficulty based on fluoroscopy times (0–50 points) [22], and Advanced LE Techniques score—to predict the necessity of using advanced extraction techniques (0–4 points) [23]. Additionally, we used our recently developed lead extraction difficulty score—LECOM score (lead dilatation time, use of second-line or advanced tools, and advanced techniques)—that turned out to be the most useful predictor of UPD (unexpected procedure difficulties) [19].

#### Leads with Their Proximal Ends Migrated into the Cardiovascular Space—Definitions

**Figure 1:**
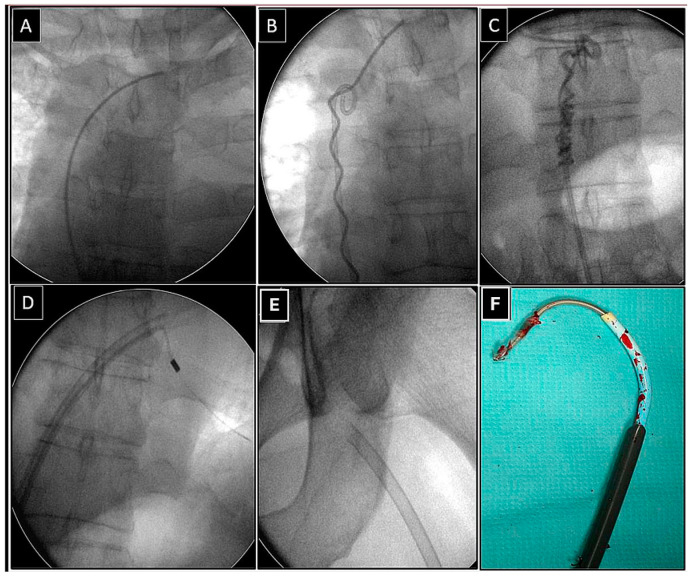
Removal of the fractured lead via the femoral approach using the “spaghetti twisting technique” to free its proximal end. The fractured bipolar ventricular lead with its proximal end in the anonymous vein (**A**). Wrapping the lead around the pig-tail catheter (**B**). Gentle traction on the pig-tail catheter releases the proximal end of the lead (**C**), which can be grasped from the femoral access and dissected (**D**). The effectively removed lead (**E**,**F**). The end of the lead was grasped with a basket catheter controlled by the curvature of the electrophysiological sheath and dissected with the rotation of a 13F bevelled Teflon catheter.

**Figure 2:**
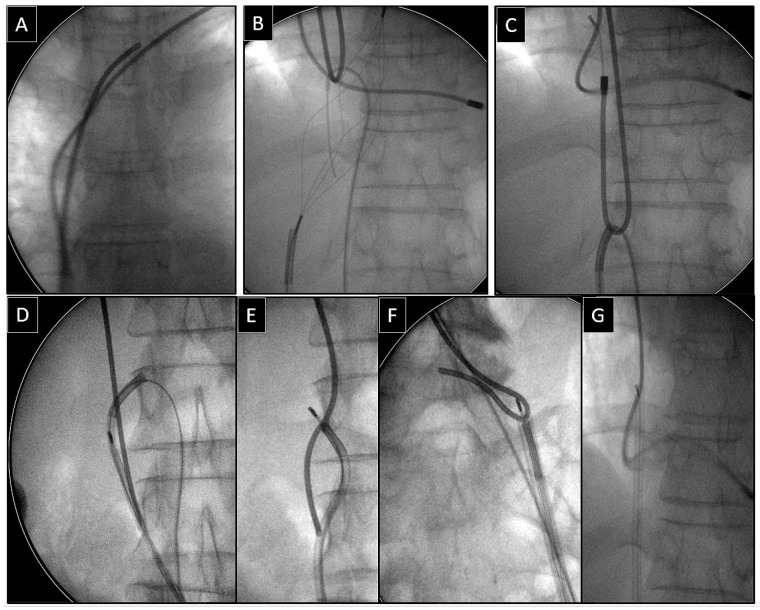
Removal of the fractured lead via the femoral access using a loop to free its proximal end. The end of the spontaneously fractured bipolar atrial lead in the anonymous vein (**A**). The loop was formed using a basket catheter controlled by a catheter for implantation of left ventricular leads and an angiographic guidewire (**B**). Pulling down the loop releases the proximal end of the lead and transfers it to the inferior vena cava (**C**,**D**). Grasping the proximal end of the lead with the basket catheter (**E**,**F**) and effective dissection of the lead using an extra-long polypropylene sheath (**G**).

**Figure 3:**
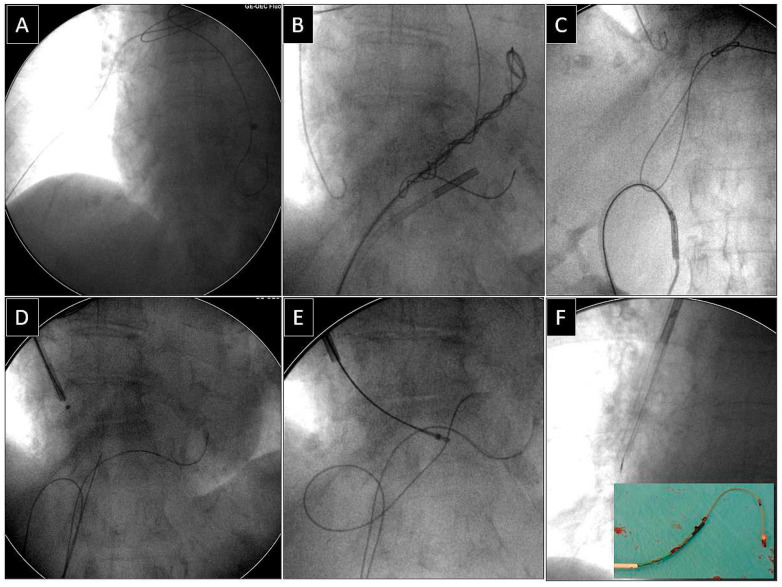
Removal of the fractured unipolar ventricular lead with its proximal end in the distal branch of the pulmonary artery, using a combined approach as well as the “spaghetti twisting technique” and a loop to release its proximal end. The fractured unipolar ventricular lead ends in the distal branch of the right pulmonary artery (**A**). Incomplete retraction of the proximal lead end from the pulmonary artery. Complicated procedure, visible backup subclavian and femoral access (the catheter for implantation of left ventricular leads acts as a femoral workstation (**B**). Effective removal of the lead from the pulmonary artery using a loop made of a lasso catheter and an angiographic guidewire (**C**,**D**). Grasping the proximal lead end through a lasso catheter (also controlled by the catheter for left ventricular leads) from the subclavian access (**E**). Conventional lead dissection using a polypropylene sheath from the subclavian approach (**F**).

**Figure 4:**
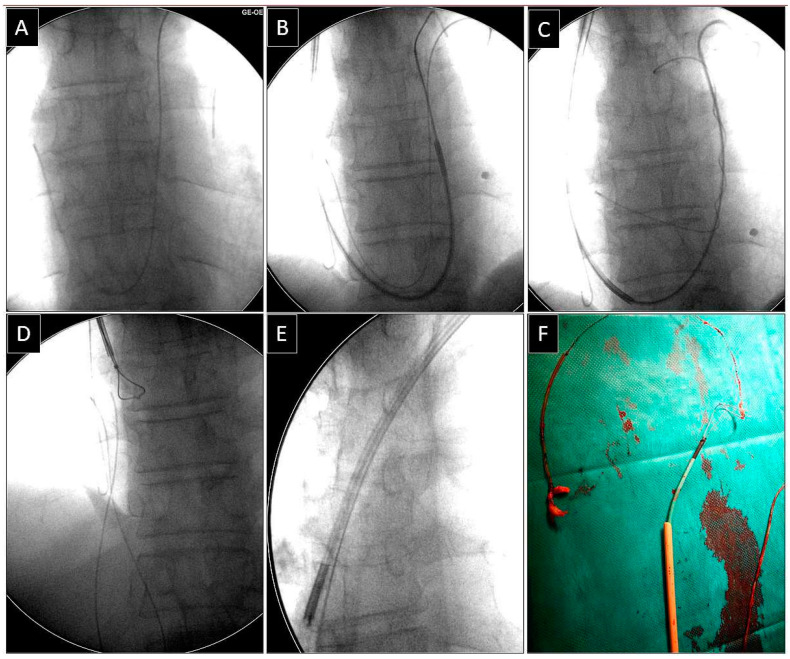
Removal of the fractured unipolar atrial lead with its proximal end in the distal branch of the pulmonary artery from the superior (subclavian) approach using the “spaghetti twisting technique” to free its proximal end. The fractured unipolar atrial lead ends in the distal branch of the left pulmonary artery (**A**). Grasping the lead in the pulmonary artery using the “spaghetti twisting technique” (**B**,**C**). The catheter dedicated to the implantation of left ventricular leads acts as a workstation, facilitating the insertion of tools into the pulmonary trunk (**B**,**C**). Successful removal of the lead from the pulmonary artery (**D**). Grasping the proximal end of the lead by a lasso catheter (also controlled by the catheter dedicated to the implantation of left ventricular leads) from the subclavian approach (**D**,**E**). Conventional lead dissection using a polypropylene catheter from the subclavian access (**E**,**F**).

**Figure 5:**
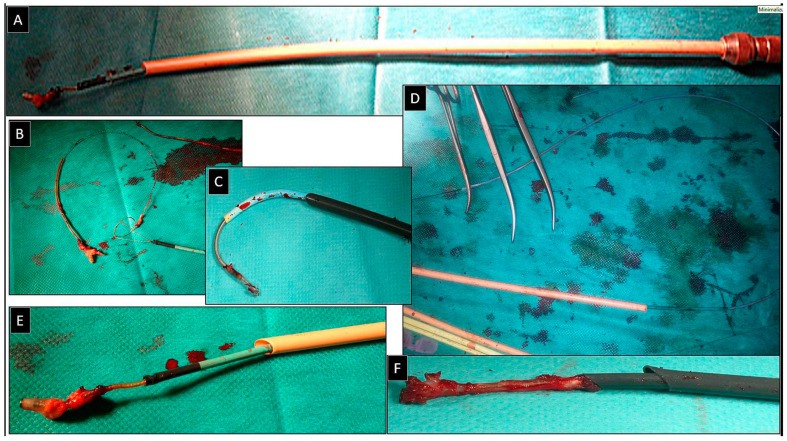
The most commonly used tools for the removal of spontaneously fractured leads with their proximal end in the cardiovascular space are lassos or basket catheters, guided by a catheter for implantation of left ventricular leads, or curved electrophysiological catheters. The fractured lead and a lasso catheter or a basket catheter act as its extension, and the sheath is an additional rail for a polypropylene or Teflon catheter. A classic set of instruments is used for the superior or femoral approach (**A**). The curved catheter for implanting left ventricular leads facilitates steering the lasso (**B**). Such a catheter allows the continuation of the procedure by dissection of the distal segment of the lead from the superior (jugular, subclavian) or femoral approach (**C**). Several surgical instruments prevent the slipping of the lead grasped by the lasso catheter (tools relieve the operator from controlling the closure of the lasso (**D**). Removed leads and tools used for dissection (**E**,**F**).

**Figure 6:**
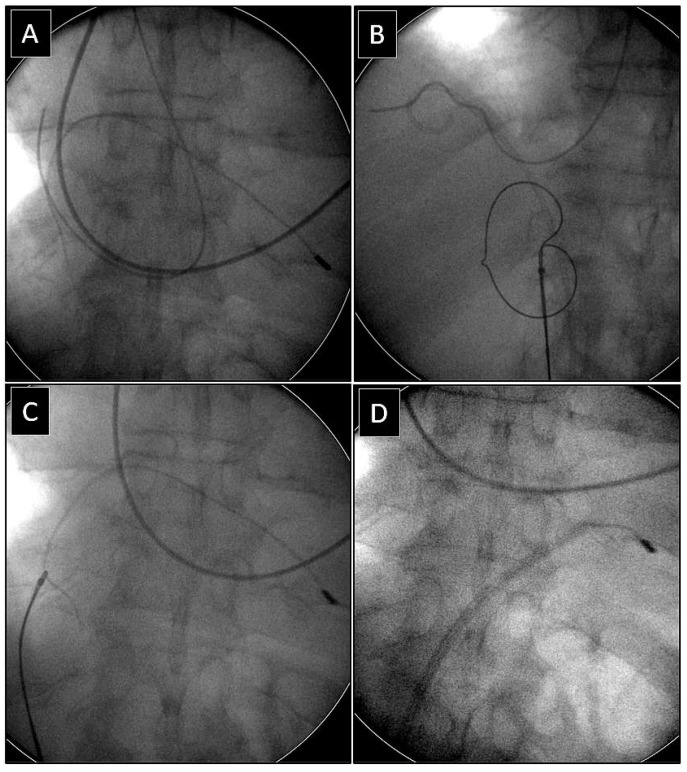
Removal of the fractured ventricular lead from the femoral approach using the “spaghetti twisting technique” to free its proximal end encased in fibrous tissue and attached to the right atrial wall. The fractured bipolar ventricular lead with its end deep in the right atrial wall (**A**). Wrapping the lead around the pig-tail catheter (**B**). Pulling on the pig-tail catheter releases the proximal end of the lead (**C**), which can be grasped with a lasso catheter (**B**,**D**), and completion of the procedure by dilation of the distal lead end with an extra-long polypropylene sheath.

LMPE were defined as fractured leads, for various reasons and via multiple mechanisms, in lead implant veins, when their proximal ends spontaneously became dislodged into the cardiovascular space and further migrated from the subclavian vein to the superior vena cava, right atrium, right ventricle to the pulmonary artery or, less frequently, to other veins, while the distal ends (lead tips) remained at the lead implant site [1,2,3,4,5,10,11,12,13,14,15] (Figure 1, Figure 2, Figure 3, Figure 4, Figure 5 and Figure 6).

### 2.3. Procedure Information

We used a stepwise approach in all patients. Standard stylets or locking stylets (Liberator Locking Stylet, Cook Medical Inc., Bloomington, IN, USA) were used, the latter ones for extraction of the oldest leads, especially passive-fixation leads, with a high estimated fracture risk. We usually commenced with non-powered mechanical telescoping polypropylene sheaths (Byrd Dilator Sheaths, Cook Medical Inc., Bloomington, IN, USA) of all sizes and lengths. Second-line tools were powered mechanical sheath systems (Evolution Mechanical Dilator Sheath, Cook Medical, Bloomington, IN, USA; TightRail Rotating Dilator Sheath, Phillips, Colorado Springs, CO, USA) or metal sheaths if the obstruction was encountered in the lead implant vein and proximal section of the subclavian vein. An alternative or combined approach using two or more different access sites (cervical, subclavian, femoral) was selected when conventional methods were found unsuccessful (LMPE or rupture of the targeted lead) [1,2,3,4,5,10,11,12,13,14,15].

#### Extraction of Leads with Migrated Proximal Ends into the Cardiovascular Space

We always performed our best to remove every LMPE. Such leads were regarded as abandoned non-functional leads and a potential source of adverse consequences of lead migration (thrombosis, venous occlusion, arrhythmias) and adverse or potentially unfavourable consequences of lead looping in the heart (TV dysfunction, arrhythmias). One of these consequences is accelerated adhesion of the lead loop to the venous wall, which may significantly hinder the removal of such leads in the event of future infectious complications [1,2,11,13,16,17,18].Thus, all such leads were treated as “any lead that, if left in place, may pose an immediate threat to the patient or any lead that, if left in place, may pose a potential threat to the patient in the future” [17,18].

Depending on the position of the proximal end of the fractured and migrated lead, we tried to retrieve it with a lasso or a basket catheter using the femoral, less often jugular approach, or, if other leads were planned for extraction, we sometimes tried to use the subclavian access re-established after removal of the other lead [1,2,3] (Figure 1, Figure 2, Figure 3, Figure 4, Figure 5 and Figure 6). After firmly grasping the proximal end of the migrated lead, the lasso or basket catheter acted as an extension of the migrant lead, and we performed lead dissection until it was removed using polypropylene sheaths or 18F bevelled catheters acting as polypropylene conventional catheters [1,2,12,13] (Figure 1 and Figure 4). Mechanical rotational tools were rarely used in our patients as most (Figure 3 and Figure 4) of these procedures had been performed before the tools became available in the market. We rarely used the Eye of the Needle Snare©, Cook Medical Inc. Bloomington, IN, USA) other techniques proved more effective. When the proximal end of the fractured migrated lead could not be grasped, it was freed from the fibrous tissue by wrapping the lead around a pig-tail catheter (“spaghetti twisting technique”) [1,2,12,13] (Figure 1, Figure 3 and Figure 4) or equally frequently using a loop made of a guidewire and a lasso catheter or basket placed over the targeted lead and pulled [1,2,9,10,14,15] (Figure 2) if the “spaghetti twisting technique” was ineffective.

Laser sheaths were not used. The organization of lead extraction has evolved in the last 17 years from procedures performed in the electrophysiology laboratory using intravenous analgesia/sedation to procedures in the hybrid room under general anaesthesia only [24]. Over the last 7 years, the core extraction team has consisted of the same highly experienced extractor (now often acting as a proctor), an experienced echocardiographer, and a dedicated cardiac surgeon [24].

### 2.4. Dataset and Statistical Methods

#### 2.4.1. Creation of Subgroups for Analysis

For the purpose of this study, 72 patients admitted for the extraction of leads with their proximal ends spontaneously migrated into the cardiovascular space, which were unavailable in the generator pocket and at the site of lead entry, were selected from the entire database for further analysis. The 72 patients with ages ranging from 13 to 88 years and a mean age of 63.42 years; 27 (37.50%) patients were women.

Since the choice of the technique of removing migrant leads depends largely on the position of the proximal end, we identified four subgroups of patients based on the locations of the proximal ends: 1. superior vena cava and right atrium (39 patients); 2. subclavian and anonymous vein (13 patients); 3. right ventricle (13 patients); 4. pulmonary artery (7 patients).

#### 2.4.2. Statistics

Continuous variables are presented as the mean ± standard deviation. The categorical variables are presented as counts and percentages. The significance of differences between the groups was determined using the non-parametric Chi^2^ test with Yates correction or the unpaired Mann–Whitney U test, as appropriate. Survival analysis was performed using the log-rank test. A *p*-value less than 0.05 was considered statistically significant. Statistical analysis was performed with Statistica 13.3 (TIBCO Software Inc., Tulsa, OK, USA).

#### 2.4.3. Approval of the Bioethics Committee

All patients gave their informed written consent to undergo TLE and use anonymous data from their medical records, approved by the Bioethics Committee at the Regional Chamber of Physicians in Lublin no. 288/2018/KB/VII (approval date: 27 November 2018). The study was carried out following the ethical standards of the 1964 Declaration of Helsinki.

## 3. Results

Since the study deals with the extraction of LMPE and the methods and difficulties in removing such leads are related to the position of the proximal end and the degree of fibrous encapsulation around the lead and its proximal end, we compared four groups of patients with different LMPE positions.

The study groups, differing in the position of the tip of the migrated lead, were comparable in terms of patient-related risk factors (age during TLE and at first system implantation, sex, heart performance, and indications), system-related risk factors (lead burden) but not in the presence of lead loops in the heart. It is obvious that the deeper the proximal end of the lead migrates into the CVS, the greater the chance of lead looping in the heart (Table 1).

The values of predicted procedure difficulty scores (which do not take into account such a relatively rare phenomenon as the lead migrated into the CVS) basically (except the MB score) did not differ between the groups, confirming their limited value in patients undergoing extraction of such leads.

Similarly, no differences were found in the procedure-related risk factors for extraction complexity, with the exception of a more frequent extraction of leads with abnormal loops in the heart when the end of the lead became dislodged deeper (into the ventricle and pulmonary artery) (Table 1).

Next, we tried to find out which leads and which models (purpose, construction) are more likely to fracture and spontaneously migrate into the CVS (Table 2).

Ventricular leads (which were also slightly more numerous than the atrial ones in the entire population) were slightly more likely to be fractured and spontaneously migrate into the CVS. However, it is clearly visible that passive-fixation leads, most often unipolar and passive bipolar, are prone to fracture and migration. Other lead models, such as BP active fixation, VDD, ICD passive, and active fixation, rarely are fractured and migrated into the CVS. Unipolar leads, thinner and more flaccid, are more likely to be rolled up in the ventricle. Bipolar leads, slightly stiffer, often retain the proximal end in the venous system, right atrium, or float to the pulmonary artery.

The two tables below summarize the most important results obtained from the present analysis. Table 3 compares the difficulty, complexity, complications, and long-term survival in patients with different positions of the tip of the lead migrated into the CVS. Table 4 provides an overview of the standard and auxiliary techniques and tools used during extraction.

Analysis of the three access sites (superior, combined, and femoral approach) showed that only in the case of leads with their proximal ends in the pulmonary artery the superior approach (re-established after removal of the other lead) was used much more often than the femoral one (71.43% vs. 14.29%).

The rate of unexpected procedure difficulties was higher, and global lead extraction time was longer if the proximal end of the targeted lead was in the pulmonary artery. Because of the rare occurrence and the relatively small number of patients in the subgroups, differences in the incidence of major complications were not significant.

Generally, the percentages of effectively removed fractured migrant leads (success) and significantly shortened lead remnants (<4 cm) were high and satisfying (93.06% and 1.39%), and the rate of complete and partial success did not differ between the study groups.

The femoral approach (45; 62.50%) was most often used for the removal of leads with their proximal ends migrated into the vasculature, whereas the superior (subclavian, jugular) approach (17; 23.61%) and combined approach (8; 11.11%) were used less frequently.

Dissection of the targeted lead using the rotation of polypropylene sheaths, 13F curved sheaths, or transseptal catheters with a bevelled end was necessary in 36 cases (51.39%) via the femoral approach and in 21 cases (29.17%) via the superior approach. Dissection was unnecessary in nine patients (12.50%) when the procedure was performed using the femoral approach and in four patients (5.56%) when superior access was used. In conclusion, lead dissection was necessary in 80.56%, whereas grasping and traction were effective in 18.06%.

The most important thing in removing an encapsulated lead with no end available in the subclavian region—i.e., the one that was retracted into the CVS, especially if it requires dissection, is to grasp its proximal or near-proximal end. As shown in the table, only 9 (12.50%) endings were free and could be grasped effectively and firmly. In the remaining patients, auxiliary tools and different approaches had to be used. The lead with a slightly encapsulated end of the lead held against the CVS wall by the elastic force usually could be moved, such that it was possible to grasp it with a pig-tail catheter in 4 (5.56%) patients.

When the lead was encased in fibrous tissue, it was freed by wrapping the lead around a pig-tail catheter (“spaghetti twisting technique”) followed by pulling downward if the manoeuvre was performed from the femoral access (8 patients, 11.11%) or pushing downward if the manoeuvre was performed from the superior access (4 patients, 5.56%). This manoeuvre was especially useful for releasing leads with their tips in the distal branch of the pulmonary artery. In a large proportion of patients (48/72, 66.67%), the proximal end of the migrated lead was strongly attached to the wall, and the above-mentioned manoeuvres did not result in its release. A loop (consisting of a guidewire and a lasso or basket catheter) was then used, and the lead was released by traction on the loop.

## 4. Discussion

Leads with their proximal ends migrated into the cardiovascular space are considered risk factors for secondary complications such as thrombosis and venous occlusion, pulmonary embolism [1,2], lead-related tricuspid valve dysfunction [1], ventricular arrhythmias [7,19], infections [1,2,13,15] and even interference with the functioning ICD devices [11,16]. Removal of migrant leads requires the use of techniques other than those used during conventional lead extraction via the lead implant vein. Case reports [5,6,7,8,9,10,11,12,13,14,15] cannot serve as a basis for assessment of the effectiveness of the methods used because only successful cases are described.

The findings of the present study show that passive-fixation pacing leads, especially unipolar, are the most common leads that are fractured and migrate into the CVS as they are slenderer and more likely to be rolled up in the ventricle. Procedure complexity in patients with such leads is high, especially with leads migrated to the pulmonary artery. However, the effectiveness of removing such leads is satisfying (93.01% and 1.4% of complete and partial success) irrespective of the position of the proximal end of the lead in the CVS.

The management of leads with their proximal ends in the cardiovascular space has been described in numerous case reports [5,6,7,8,9,10,11,12,13,14,15], case series [3], and a few studies [1,2]. The following factors play a key role: venous access allowing the insertion of tools to grasp the targeted lead, freeing its proximal end from fibrous tissue, effectively grasping the end, and then removing the entire lead by traction, counter-traction, or dissection of the lead from the fibrous tissue if necessary [1,2,3].

Literature shows that the femoral approach is most common [3,10,12,13,14] whereas the superior approach, i.e., subclavian or jugular, is slightly less frequently selected [1,2,3]. Combined access is defined as a situation in which it is necessary to use two access sites simultaneously to remove the lead; it has been described relatively rarely [1,11,14]. In the present study, the femoral approach was most common (63.89%), whereas the superior (23.62%) and the combined approach (11.11%) were used less frequently. Overall, 80.56% of leads needed mechanical dilation regardless of the access used. Lead dilatation was unnecessary in 18.06% of procedures; a grab and a simple pull-out were enough.

Because of a prolonged dwell time, the end of the fractured lead is sometimes firmly attached to the walls and needs to be released using special techniques and auxiliary tools to pull the end of the lead out of the scar. These auxiliary tools include a loop formed over the lead [1,2,12,13,14,15] or a pig-tail catheter on which the lead is wound (“spaghetti twisting technique”) [1,2,3,14]. These instruments can be inserted from the superior [1,3,8] or femoral approach [1,4].

In this study, in 66.7% of procedures, the migrated proximal end of the spontaneously fractured lead was encased in fibrous tissue, and a loop (consisting of a guidewire and a lasso or basket catheter) had to be applied to release the end by traction.

Sometimes (in 10.8% of procedures), wrapping the lead around a pig-tail catheter (“spaghetti twisting technique”) followed by pushing or pulling downward, depending on the access site used, was sufficient to free the lead end. This manoeuvre was especially useful for releasing leads with their ends in the distal branch of the pulmonary artery. All these manoeuvres were performed from the femoral or superior approach, depending on the anatomical conditions. Only 12.5% of migrated proximal ends of fractured leads were free and could be grasped effectively and firmly.

We did not find an association between the need to use specific tools and methods for the extraction of leads with migrated proximal ends and long-term mortality. To the best of our knowledge, no one has described in detail the methods, tools, and effectiveness of removing the leads with their proximal ends migrated into the cardiovascular space. It appears that techniques for extraction of leads with proximal ends migrated into the CVS (LMPEs) should be included in the TLE training curriculum. Removal of such leads requires a high level of operator experience, the availability of a wide range of tools, and the possibility of assistance from an interventional radiologist.

## 5. Conclusions

1. Spontaneous lead fractures with their proximal ends migrated into the cardiovascular space are a rare finding among patients referred for transvenous lead extraction (1.87%).
2. Removal of such leads requires the use of different approaches and dedicated and non-dedicated tools.
3. In spite of a high level of procedure complexity, its effectiveness is equally high (93.06% procedural success) with an acceptable rate of major complications.

## 6. Study Limitations

There are some limitations in this study. It presents the experience of three centres but the same first operator who is now acting as a proctor. Data were prospectively collected but analysed retrospectively. Procedures were performed using all types of mechanical systems but not laser-powered sheaths. An important limitation in the present study is the selection of patients, and this population does not represent all patients with fractured leads.

A certain minor limitation in the present study is the fact that some of the case studies mentioned in the Discussion were previously described by investigators from other national centres. The study analyses a relatively large population of patients with a rare occurrence of spontaneous lead fracture with an implant duration longer than in many recent studies because our centre for many years had been an unofficial reference centre, and we were receiving the most difficult patients in the country. This explains the rate of major complications and a lower rate of radiographic success. Our experiences should be of interest to all those who will face extraction of old passive-fixation leads and management of less frequent lead-related permanent pacing complications.

The last one is a presentation of a single, experienced first operator and his team. The results are probably not repeatable by even a moderately experienced operator in a low-volume centre, especially with respect to leads with long or very long duration and MPE leads.

## 7. Impact on Daily Practice

Spontaneous lead fracture, an unusual complication, can cause migration of its proximal end into the vascular space, frequently with subsequent looping in the heart. Removal of such leads requires the use of less standard techniques and less conventional auxiliary tools. This report presents a broad range of approaches and the effectiveness of dedicated and non-dedicated tools for the removal of fractured leads. To the best of our knowledge, this is the first paper addressing this important problem.

## Figures and Tables

**Table 1:** Patient-, system- and procedure-related risk factors.

Position of the proximal end of the fractured lead that migrated into CVS					
Patient-, system- and procedure-related risk factors	Superior vena cava and right atrium	Subclavian and anonymous vein	Right ventricle	Pulmonary artery	The sum of the four groups listed
Number of patients/group number	Group 1 N = 39 Mean ± SD N (%)	Group 2 N = 13 Mean ± SD N (%)	Group 3 N = 13 Mean ± SD N (%)	Group 4 N = 7 Mean ± SD N (%)	Group 5 N = 72 Mean ± SD N (%)
		*p*: 2 vs. 1	*p*: 3 vs. 1	*p*: 4 vs. 1	
Patient-related risk factors					
Patient age during TLE [years]	64.31 ± 15.97	62.15 ± 20.04 *p* = 0.695	64.54 ± 7.88 *p* = 0.960	58.71 ±16.50 *p* = 0.400	63.42 ± 15.53
Patient age at first system implantation [years]	51.39 ± 18.42	50.92 ± 17.83 *p* = 0.937	49.62 ± 11.02 *p* = 0.684	46.43 ± 15.70 *p* = 0.508	50.50 ± 16.70
Female	15 (38.46)	5 (38.46) *p* = 0.742	5 (38.46) *p* = 0.742	2 (28.57) *p* = 0.941	27 (37.50)
LVEF, average [%]	53.52 ± 13.66	59.54 ± 11.41 *p* = 0.345	48.77 ± 17.17 *p* = 0.314	57.29 ±14.24 *p* = 0.507	54.22 ± 14.16
Infectious indications	9 (23.08)	5 (38.46) *p* = 0.470	5 (38.46) *p* = 0.470	3 (42.86) *p* = 0.529	22 (30.56)
System-related risk factors					
Number of leads in the heart before TLE					
1 or 2	20 (51.28)	9 (69.23) *p* = 0.402	9 (69.23) *p* = 0.402	3 (42.86) *p* = 1.000	41 (56.94)
3 and more	19 (48.72)	4 (30.77) *p* = 0.402	4 (30.77) *p* = 0.402	4 (57.14) *p* = 1.000	31 (43.06)
Lead loops in the heart					
Long lead loop interfering with tricuspid apparatus	16 (41.03)	5 (38.46) *p* = 0.870	11 (84.62) *p* = 0.016	7 (100.0) *p* = 0.014	39 (54.17)
Short lead loop in the heart	23 (58.97)	7 (53.85) *p* = 1.000	2 (15.38) *p* = 0.016	0 (0.00) *p* = 0.014	32 (44.44)
Predicted procedure difficulty					
MB score, average [need for advanced tools] [21] [points]	2.92 ±1.31	3.23 ± 0.93 *p* = 0.436	3.81 ± 0.95 *p* = 0.029	3.57 ± 0.98 *p* = 0.358	3.11 ± 1.16
LED index (predicted fluoroscopy time) [22] [points]	14.26 ± 7.98	13.23 ± 4.09 *p* = 0.695	16.23 ± 5.57 *p* = 0.423	14.86 ± 4.85 *p* = 0.848	14.49 ± 6.71
Advanced TLE (ALET-Mazzone) scale (need for advanced TLE techniques) [23] [points]	2.18 ± 0.91	2.15 ± 0.80 *p* = 0.939	2.23 ± 0.83 *p* = 0.875	2.71 ± 1.11 *p* = 0.274	2.24 ± 0.90
LECOM score (predicted procedure complexity) [points] [19]	12.92 ± 5.27	12.58 ± 4.51 *p* = 0.834	14.14 ± 5.49 *p* = 0.478	14.55 ± 3.18 *p* = 0.435	13.23 ± 4.97
Lead or lead loop interfering with tricuspid apparatus or pulmonary valve	16 (41.03)	5 (38.46) *p* = 0.870	11 (84.62) *p* = 0.016	7 (100.0) *p* = 0.014	39 (54.17)
Procedure-related risk factors					
Extraction of multiple leads (≥3)	11 (28.21)	3 (23.08) *p* = 1.000	1 (7.69) *p* = 0.254	4 (57.14) *p* = 0.286	19 (26.39)
Extraction of abandoned leads	23 (58.97)	8 (1.54) *p* = 0.870	10 (76.92) *p* = 0.406	6 (85.71) *p* = 0.355	47 (65.28)
Extraction of leads with too much slack in the heart	17 (43.59)	5 (38.46) *p* = 1.000	12 (92.31) *p* = 0.006	7 (100.0) *p* = 0.019	41 (56.94)
Oldest extracted lead per patient [months]	149.1 ± 89.01	138.5 ± 48.92 *p* = 0.683	176.3 ± 67.80 *p* = 0.319	149.2 ± 65.70 *p* = 0.998	152.2 ± 77.03
Average lead dwell time per patient [months]	21.43 ± 16.93	18.25 ± 9.94 *p* = 0.526	23.22 ± 12.81 *p* = 0.729	25.94 ± 16.29 *p* = 0.518	21.62 ± 14.99

CVS—cardiovascular space, N—number, TLE—transvenous lead extraction, MB score (need for advanced tools), LED index (predicted procedure fluoroscopy time), Advanced TL (ALET—need for advanced TLE techniques), LECOM score (predicted procedure complexity).

**Table 2:** Information on fractured and migrated leads.

Position of proximal ends of fractured leads migrated into CVS					
Fractured lead information	Superior vena cava and right atrium	Subclavian and anonymous vein	Right ventricle	Pulmonary artery	The sum of the four groups listed
Number of patients/group number	Group 1 N = 39 Mean ± SD N (%)	Group 2 N = 13 Mean ± SD N (%)	Group 3 N = 13 Mean ± SD N (%)	Group 4 N = 7 Mean ± SD N (%)	Group 5 N = 72 Mean ± SD N (%)
		*p*: 2 vs. 1	*p*: 3 vs. 1	*p*: 4 vs. 1	
Fractured lead dwell time [months]	141.5 ± 82.41	138.5 ± 48.92 *p* = 0.902	175.9 ± 83.04 *p* = 0.198	149.0 ± 68.62 *p* = 0.844	148.0 ± 76.05
Position of fractured lead tips					
Right ventricle	20 (51.28)	7 (53.85) *p* = 0.873	9 (69.23) *p* = 0.420	5 (71.43) *p* = 0.566	41 (56.94)
Right atrium	17 (43.59)	5 (38.46) *p* = 1.000	4 (30.77) *p* = 0.625	2 (28.57) *p* = 0.743	28 (38.89)
Other (CS)	2 (5.13)	1 (7.69) *p* = 0.731	0 (0.00) *p* = 1.000	0 (0.00) *p* = 0.694	3 (4.17)
Model of the fractured lead					
UP passive fixation	12 (30.77)	4 (30.77) *p* = 0.729	11 (84.62) *p* = 0.002	3 (42.86) *p* = 0.849	30 (41.67)
BP passive fixation	25 (64.10)	8 (61.54) *p* = 0.868	1 (7.69) *p* = 0.001	3 (42.86) *p* = 0.522	37 (51.39)
BP active fixation	1 (2.56)	0 (0.00) *p* = 0.560	0 (0.00) *p* = 0.560	0 (0.00) *p* = 0.378	1 (1.39)
VDD passive fixation	0 (0.00)	0 (0.00) N	1 (7.69) *p* = 0.560	0 (0.00) *p* = 0.378	1 (1.39)
ICD passive fixation	1 (2.56)	1 (7.69) 1.000	0 (0.00) *p* = 0.560	1 (14.29) *p* = 0.694	1 (1.39)
ICD active fixation	0 (0.00)	0 (0.00) NC	0 (0.00) NC	0 (0.00) NC	0 (0.00)

CVS—cardiovascular space, N—number, NC—non-comparable, CS—coronary sinus, UP—unipolar, BP bipolar, VDD—atrial sensing, ventricular sensing/pacing lead, ICD—implantable cardioverter defibrillator.

**Table 3:** Extraction procedure information (difficulty, complexity, complications) and long-term survival.

The position of the proximal end of the fractured lead migrated into CVS					
TLE procedure information	Superior vena cava and right atrium	Subclavian and anonymous vein	Right ventricle	Pulmonary artery	The sum of the four groups listed
Number of patients/group number	Group 1 N = 39 Mean ±SD N (%)	Group 2 N = 13 Mean ± SD N (%)	Group 3 N = 13 Mean ± SD N (%)	Group 4 N = 7 Mean ± SD N (%)	Group 5 N = 72 Mean ± SD N (%)
		*p*: 2 vs. 1	*p*: 3 vs. 1	*p*: 4 vs. 1	
Methods of removing fractured migrant leads					
Superior approach	6 (15.39)	3 (23.08) *p* = 0.832	3 (23.08) *p* = 0.832	5 (71.43) *p* = 0.007	17 (23.61)
Combined approach	3 (7.69)	3 (23.08) *p* = 0.316	1 (7.67) *p* = 0.548	1 (14.29) *p* = 0.874	8 (11.11)
Femoral approach	29 (74.36)	7 (53.85) *p* = 0.298	8 (61.54) *p* = 0.596	1 (14.29) *p* = 0.001	45 (62.50)
Removed during cardiac surgery	1 (2.56)	0 (0.00) *p* = 0.560	1 (7.67) *p* = 1.000	0 (0.00) NC	2 (2.78)
Procedure difficulty and complexity					
Number of unexpected procedure difficulties	1.47 ± 0.84	1.39 ± 0.65 *p* = 0.744	1.55 ± 0.82 *p* = 0.777	2.43 ± 0.79 *p* = 0.007	1.43 ± 0.76
Two or more unexpected procedure difficulties	10 (25.64)	4 (30.77) *p* = 1.000	4 (30.77) *p* = 1.000	5 (71.43) *p* = 0.052	18 (25.00)
CID-TLE score (1–5 points) average	3.80 ± 0.52	4.08 ± 0.49 *p* = 0.094	3.77 ± 0.73 *p* = 0.888	3.86 ± 0.69 *p* = 0.802	3.85 ± 0.57
CID-TLE score 4 and > points	31(79.49)	12 (92.31) *p* = 0.526	10 (76.93) *p* = 0.845	5 (71.43) *p* = 0.983	58 (89.56) *p* = 0.
Global lead dilatation time	83.54 ± 54.08	61.62 ± 29.43 *p* = 0.171	65.77 ± 19.67 *p* = 0.254	105.7 ± 46.50 *p* = 0.003	78.53 ± 46.14
Single lead dilatation time (minutes)	48.24 ± 27.82	38.57 ± 18.38 *p* = 0.249	43.08 ± 15.18 *p* = 0.528	43.21 ± 14.05 *p* = 0.644	45.03 ± 23.19
Major complications	2 (5.13)	0 (0.00) *p* = 1.000	0 (0.00) *p* = 1.000	0 (0.00) *p* = 0.694	2 (2.78)
Haemopericardium	1 (2.56)	0 (0.00) *p* = 0.560	0 (0.00) *p* = 0.560	0 (0.00) *p* = 0.328	1 (1.39)
Haemothorax	0 (0.00)	0 (0.00) NC	0 (0.00) NC	0 (0.00) NC	0 (0.00)
Rescue cardiac surgery	1 (2.56)	0 (0.00) *p* = 0.560	0 (0.00) *p* = 0.560	0 (0.00) *p* = 0.694	1 (1.39)
Final effect of lead remnant extraction/removal					
Effective remnant removal (success)	35 (89.74)	13 (100.0) *p* = 0.548	12 (92.31) *p* = 0.786	7 (100.0) *p* = 0.874	67 (93.06)
Reduction in lead remnant length (<4 cm)	1 (2.56)	0 (0.00) *p* = 0.560	0 (0.00) *p* = 0.560	0 (0.00) *p* = 0.328	1 (1.39)
Ineffective attempt or removal	1 (2.56)	0 (0.00) *p* = 0.560	0 (0.00) *p* = 0.560	0 (0.00) *p* = 0.328	1 (1.39)
Planned or rescue cardiac surgery	2 (5.13)	0 (0.00) *p* = 1.000	1 (7.69) *p* = 1.000	0 (0.00) *p* = 0.694	3 (4.17)
Long-term mortality					
Survivors	22 (56.41)	8 (61.54) *p* = 1.000	8 (61.54) *p* = 1.000	6 (85.71) *p* = 0.297	44 (61.11)
Death within one year after TLE	4 (10.26)	2 (15.39) *p* = 1.000	2 (15.38) *p* = 1.000	1 (14.29) *p* = 0.659	9 (12.50)
Death at > 1 year after TLE	13 (33.33)	3 (23.08) *p* = 0.729	3 (23.08) *p* = 0.729	0 (0.00) *p* = 0.179	19 (26.39)

CVS—cardiovascular space, N—number, TLE—transvenous lead extraction, CID-TLE—Complex Indicator of Difficulty of TLE, NC—non-comparable,.

**Table 4:** Vascular access, techniques, and tools used for removal of LMPEs.

Superior Approach	N (%)	Combined Approach	N (%)
Main/final approach and tools	17 (100.0)	Main/final approach and tools	8 (100.0)
Lasso/basket in CS sheath and a polypropylene or rotational sheath over them (dilatation)	13 (76.47)	Lasso/basket in CS sheath and a polypropylene rotational sheath over them—superior approach (dilatation)	8 (100.0)
Lasso/basket in CS sheath—superior (pulling only)	1 (5.88)	Lasso/basket in CS sheath—superior approach (pulling only)	0 (0.00)
Lasso/basket only (pulling only)	3 (17.65)	Lasso/basket only—superior approach (pulling only)	0 (0.00)
Supporting tools and techniques	17 (100.0)	Supporting tools and techniques	8 (100.0)
Loop (pulling, end release)	6 (35.29)	Loop femoral approach	6 (75.00)
Pig-tail + winding and shifting superior approach (end release)	3 (17.65)	Pig-tail femoral approach	1 (12.50)
Pig-tail superior (end orientation)	0 (0.00)	Pig-tail + winding and shifting femoral approach	1 (12.50)
Not used	8 (47.06)	Not used	0 (0.00)
**Femoral Approach**		**All Migrated Lead Removal**	
Main/final approach and tools	45 (100.0)	Main/final approach and tools	72 (100.0)
Lasso/basket inside 13 FWS, NE (dilatation)	31 (68.89)	Lasso/basket 13, FWS, NE—femoral approach (dilatation)	31 (43.06)
Lasso/basket inside another large sheath (polypropylene) (dilatation)	5 (11.11)		
Lasso/basket inside transseptal sheath (only pulling)	9 (20.00)	Lasso/basket another large sheath—femoral (dilatation)	5 (8.33)
		Lasso/basket in CS sheath and a polypropylene rotational sheath over them—superior approach (dilatation)	21 (29.17)
Supporting tools and techniques	45 (100.0)	Lasso/basket inside transseptal sheath—femoral approach (pulling only)	9 (12.50)
Loop femoral approach (pulling, end release)	35 (77.78)	Lasso/basket in CS sheath—superior approach (pulling only)	1 (1.39)
Pig-tail + winding and shifting femoral (end release)	7 (15.56)	Lasso/basket only—superior approach (pulling only)	3 (4.17)
Pig-tail femoral (end orientation)	3 (6.67)	Cardiac surgery	2 (2.28)
Surgical approach among 72 procedures		Supporting tools and techniques	72 (100.0)
Risk of MC-TLE procedure interrupted	1 (1.39)	Loop femoral approach (pulling, end release)	42 (58.33)
Haemopericardium—rescue cardiac surgery	1 (1.39)	Loop superior approach (pulling, end release)	6 (8.33)
All:	2 (2.28)	Pig-tail + winding and shifting femoral approach (pulling, end release)	8 (11.11)
		Pig-tail + winding and shifting superior (pulling, end release)	3 (4.17)
		Pig-tail femoral approach (end orientation)	4 (5.56)
		Pig-tail superior (end orientation)	0 (0.00)
		Not used	9 (12.50)

N—number, CS—coronary sinus, FWS—femoral workstation, MC—major complication, TLE—transvenous lead extraction.

## Data Availability

Readers can access the data supporting the conclusions of the study at www.usuwanieelektrod.pl, accessed on 12 December 2024).

## Abbreviations and Acronyms

ICD—implantable cardioverter defibrillator; CS—coronary sinus; CVS—cardiovascular space; LMPE—leads with migrated proximal ends; MC—major complication; TLE—transvenous lead extraction.
